# Infection with Non-Lethal West Nile Virus Eg101 Strain Induces Immunity that Protects Mice against the Lethal West Nile Virus NY99 Strain

**DOI:** 10.3390/v6062328

**Published:** 2014-06-06

**Authors:** Mukesh Kumar, Maile O’Connell, Madhuri Namekar, Vivek R. Nerurkar

**Affiliations:** 1 Department of Tropical Medicine, Medical Microbiology and Pharmacology, John A. Burns School of Medicine, University of Hawaii at Manoa, Honolulu, HI 96813, USA; E-Mails: mukesh@hawaii.edu (M.K.); maileo@hawaii.edu (M.O.); madhuris@hawaii.edu (M.N.); 2 Pacific Center for Emerging Infectious Diseases Research, John A. Burns School of Medicine, University of Hawaii at Manoa, Honolulu, Hawaii 96813, USA

**Keywords:** West Nile virus, flavivirus, WNV NY99, WNV Eg101, encephalitis, humoral immunity, vaccine

## Abstract

Herein we demonstrate that infection of mice with West Nile virus (WNV) Eg101 provides protective immunity against lethal challenge with WNV NY99. Our data demonstrated that WNV Eg101 is largely non-virulent in adult mice when compared to WNV NY99. By day 6 after infection, WNV-specific IgM and IgG antibodies, and neutralizing antibodies were detected in the serum of all WNV Eg101 infected mice. Plaque reduction neutralization test data demonstrated that serum from WNV Eg101 infected mice neutralized WNV Eg101 and WNV NY99 strains with similar efficiency. Three weeks after infection, WNV Eg101 immunized mice were challenged subcutaneously or intracranially with lethal dose of WNV NY99 and observed for additional three weeks. All the challenged mice were protected against disease and no morbidity and mortality was observed in any mice. In conclusion, our data for the first time demonstrate that infection of mice with WNV Eg101 induced high titers of WNV specific IgM and IgG antibodies, and cross-reactive neutralizing antibodies, and the resulting immunity protected all immunized animals from both subcutaneous and intracranial challenge with WNV NY99. These observations suggest that WNV Eg101 may be a suitable strain for the development of a vaccine in humans against virulent strains of WNV.

## 1. Introduction

West Nile virus (WNV), an enveloped, single-stranded positive-sense, neurotropic flavivirus, is an important human pathogen that causes potentially lethal encephalitis [1]. WNV was originally isolated from a febrile patient in Uganda in 1937 [2]. Until 1999, WNV was geographically distributed in Africa, the Middle East, western and central Asia, India, and Europe, where it caused sporadic cases of febrile disease and occasional outbreaks of encephalitis in elderly people and in equines [3,4,5]. The unexpected emergence of WNV in the United States in 1999 was associated with the introduction of the NY99 strain, which is more virulent, and results in higher incidence of meningoencephalitis in humans as compared to the non-virulent strains such as WNV Eg101 strain [5,6,7,8]. In the United States between 1999 and 2012, an estimated 3 million people were infected with WNV, resulting in over 780,000 clinical cases, with 16,196 neuroinvasive cases and 1549 deaths [4,9]. Recent outbreaks of highly virulent WNV strains have also been reported in the Mediterranean basin, southern Europe and Russia [10,11,12]. Although the worldwide incidence of WNV infection is increasing, there is no specific treatment or vaccine available for use in humans.

Protection against primary WNV infection or secondary challenge is linked to the induction of protective humoral and cellular immune responses. The induction of WNV-specific immunoglobulins (IgM and IgG) is essential for suppressing viremia and virus dissemination. It has been demonstrated that passive transfer of serum containing WNV-specific antibodies protects against virus dissemination into the central nervous system and prevents lethal encephalitis and death [13,14,15,16]. T cell-mediated immunity is essential for controlling WNV infection in the brain [17,18,19]. Therefore, an effective WNV vaccine should be able to mount both humoral and T cell responses and limit virus replication both in the periphery as well in the brain to achieve complete protection. Live-attenuated replicating vaccines are highly immunogenic and elicit robust adaptive immune responses [20,21]. However, the development of a live attenuated WNV vaccine, which may have a better capability to elicit balanced humoral and cell mediated immune responses, is hindered by the high virulence and pathogenicity of the WNV strains circulating in the United States. A formalin-inactivated WNV vaccine of moderate efficacy has been developed for equine immunization [22]. However, this vaccine is also generated from highly virulent NY99 strain of WNV and raises the safety concerns for immunization of humans, especially those at risk for severe disease such as the elderly and immunocompromised. An alternative to using virulent WNV NY99 strain is to develop a vaccine based using a naturally non-virulent strain, such as WNV Eg101 [5,9].

Eg101 strain of WNV is largely non-pathogenic and antigenically very closely related to the lethal WNV NY99 strain. WNV Eg101 was isolated in 1950 from normal appearing children near Cairo, Egypt [23]. Seroprevalence among adults in the Nile Delta region has been demonstrated to be 61% to WNV Eg101 with little or no evidence of disease [24]. WNV Eg101 strain has 95.4% of nucleotide and 99.6% of amino acid identity to WNV NY99 strain [8,25]. Both the WNV NY99 and WNV Eg101 strains are classified in same genotypic lineage and belong to same clade 1a of the lineage 1 [8]. Lineage 1 strains are considered emerging and associated with outbreaks of neuroinvasive disease [8]. Therefore, WNV Eg101 strain may be a suitable strain for the development of a vaccine in humans against the more virulent strains such as WNV NY99. Herein we demonstrate that infection of mice with WNV Eg101 provides protective immunity against lethal challenge including intracranial inoculation of high dose of WNV NY99. WNV Eg101 induces cross-reactive antibodies that neutralize the WNV NY99 strain with efficiency similar to that observed for neutralization of WNV Eg101.

## 2. Materials and Methods

### 2.1. Animals

Eight-week old C57BL/6J mice were purchased from The Jackson Laboratory (Bar Harbor, ME, USA). Animals were housed four per cage and allowed to eat and drink ad libitum. The animal suite was maintained at 72 °F, 45% humidity and on 12/12-light/dark cycles. Sawdust bedding was provided along with paper towels. Trained and certified personnel conducted all the animal experiments. This study was approved by the University of Hawaii Institutional Animal Care and Use Committee (IACUC), and was conducted in strict accordance with guidelines established by the National Institutes of Health and the University of Hawaii IACUC.

### 2.2. WNV Infection Experiment

Mice were inoculated via the footpad route with 1000 or 100 plaque forming units (PFU) of Eg101 or NY99 strains of WNV or PBS (Mock) and the disease symptoms and mortality were observed for 21 days as described previously [26,27,28,29]. Clinical symptoms were observed twice a day as described previously [26]. Mice that exhibited severe disease were euthanized using CO2 to limit suffering. On specific days after infection, 100 µL blood was collected from the tail vein, and serum was separated and frozen at −80 °C for further analysis.

### 2.3. WNV Plaque Assay

WNV titers in the serum were measured by plaque assay using Vero cells as described previously [26,30,31].

### 2.4. Measurement of WNV-Specific Antibodies

The titers of WNV-specific IgM and IgG antibodies were measured in the serum of WNV Eg101 infected mice using microsphere immunoassay (MIA) for WNV envelope E protein as described previously [26,32]. Briefly, serum samples (1:20 dilution) were incubated with the microspheres coupled with a recombinant WNV E antigen for 30 min followed by secondary goat anti-mouse IgM or IgG conjugated to red-phycoerythrin for 45 min. The fluorescence intensity of the microspheres was analyzed with a Luminex 100 instrument as described previously [32].

### 2.5. Plaque Reduction Neutralization Test (PRNT)

The titers of anti-WNV neutralizing antibodies were measured in the serum of WNV Eg101 infected mice using PRNT assay as described previously [32]. Serum was diluted serially from 1:40 to 1:5000 and PRNT was conducted by using Eg101 and NY99 strains of WNV, as described previously [32]. The highest dilution of serum resulting in 80% reduction in the number of plaques compared to the growth of the virus control was determined as described previously [33].

### 2.6. Challenge Experiment

Three-weeks after infection, PBS and WNV Eg101 immunized mice were challenged subcutaneously or intracranially with 1000 PFU of WNV NY99 and the disease symptoms and mortality were observed for 21 days after challenge. On specific days after challenge, 100 µL blood was collected from the tail vein, and serum was separated and frozen at −80 °C for further analysis.

### 2.7. Statistical Analysis

Log-rank (Mantel-Cox) Test and Gehan-Breslow-Wilcoxon Test were used to analyze the survival data [34]. Mann-Whitney test and unpaired student t-test were used to calculate *p* values of difference between viral titers and antibodies responses, respectively [34]. Differences of *p* < 0.05 were considered significant.

## 3. Results

### 3.1. Virulence of WNV Eg101 and WNV NY99 Strains in Eight-Week-Old C57BL/6J Mice

**Figure 1:**
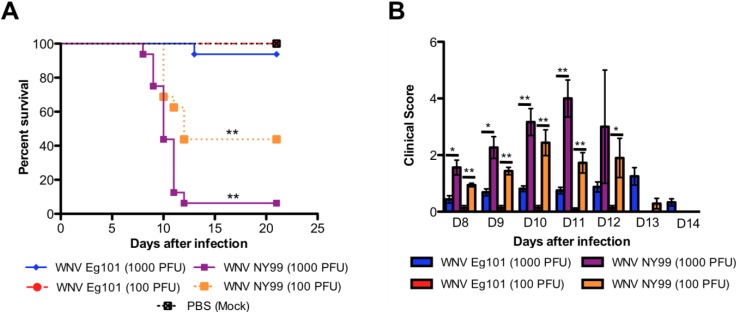
Survival analysis and clinical score of WNV Eg101 and WNV NY99 infected mice. (**A**) Eight-week old C57BL/6J mice were inoculated subcutaneously with 1000 or 100 PFU of WNV Eg101 or WNV NY99. A group of mice were also inoculated with PBS (Mock). All mice were observed for 21 days. Data are combined of two independent studies (n = 16 per group). (**B**) Animals were monitored for clinical scores twice a day. The designation for the clinical scores is as follows: 1, ruffled fur/hunched back; 2, paresis/difficulty walking; 3, paralysis; 4, moribund/euthanized; and 5, dead. Error bars represent SEM. * *p* < 0.001, ** *p* < 0.0001.

Eight-week-old C57BL/6J mice were infected subcutaneously with 1000 or 100 PFU of WNV Eg101 or WNV NY99 or PBS (Mock). PBS inoculated mice remained healthy throughout the observation period of 21 days. As expected, infection of mice with 1000 PFU of WNV NY99 was highly lethal and resulted in 94% mortality. In comparison, only 6% mortality was observed in mice infected with 1000 PFU of WNV Eg101 (Figure 1A). Similarly, mice inoculated with 100 PFU of WNV NY99 exhibited high (60%) mortality, while no mortality was observed in mice infected with 100 PFU of WNV Eg101 (Figure 1A). Mortality rates were significantly high in WNV NY99 infected mice (both 1000 and 100 PFU, *p* < 0.0001) than WNV Eg101 infected mice. All surviving animals were positive for anti-WNV IgG antibodies (data not shown).

As depicted in Figure 1B, all mice infected with both 1000 and 100 PFU of WNV NY99 demonstrated clinical evidence of infection characterized by ruffled fur and hunchbacked posture, and severe neurological symptoms such as paresis, hind limb paralysis, tremors and ataxic gait. In comparison, only 25% of mice infected with 1000 PFU of WNV Eg101 exhibited symptoms such as ruffled fur and hunchbacked posture and only one mouse demonstrated severe neurological symptoms. No clinical symptoms were observed in the mice infected with 100 PFU of WNV Eg101.

### 3.2. WNV Titers in the Serum of WNV Eg101 and WNV NY99 Infected Mice

**Figure 2:**
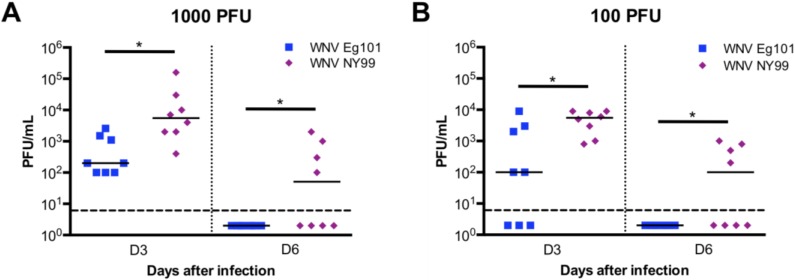
Virus titers in the serum of WNV Eg101 and WNV NY99 and infected mice. The kinetics of virus replication and levels of WNV were determined in the serum of the mice infected with (**A**) 1000 PFU, and (**B**) 100 PFU of WNV Eg101 and WNV NY99 at indicated time-points by plaque assay. The data are expressed as PFU/mL of serum. Each data point represents an individual mouse, and data from two independent experiments are depicted (n = 8 mice per group). Data points below the horizontal dotted line are negative. The solid horizontal line signifies the median of eight mice per group. * *p* < 0.05.

The WNV replication kinetics in the serum of mice was measured by plaque assay. At day 3 after infection, WNV was detected in the serum of all the mice infected with 1000 and 100 PFU of WNV NY99 (Figure 2). Similarly, WNV was also detected in the serum of all the mice infected with 1000 PFU of WNV Eg101. In comparison, only 62% of mice infected with 100 PFU of WNV Eg101 had detectable viremia at day 3 after infection. At day 6 after infection, the virus was cleared from the periphery of all the WNV Eg101 infected mice, however, it remained high in 50% of the WNV NY99 infected mice (Figure 2). The virus titers were significantly higher in WNV NY99 infected mice when compared to WNV Eg101 infected mice at both days 3 and 6 after infection.

### 3.3. Induction of WNV-Specific Antibodies Responses in WNV Eg101 Infected Mice

**Figure 3:**
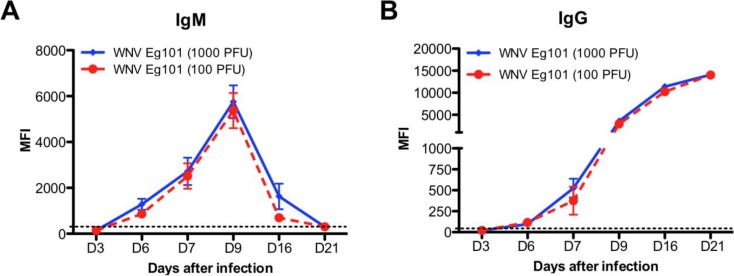
WNV-specific IgM and IgG antibodies titers in the serum of WNV Eg101 infected mice. Serum was collected from WNV Eg101 infected mice at indicated time points and WNV-specific (**A**) IgM and (**B**) IgG responses were measured by MIA using WNV E antigen as described in materials and methods. Results are reported as median fluorescent intensity (MFI) per 100 microspheres. Data are expressed as MFI ±SEM and is representative of two independent experiments (n = 8–16 mice per group). Dotted line indicates the cutoff value.

The titers of WNV-specific IgM and IgG antibodies in the serum of Eg101 infected mice were measured using MIA. Infection with both 1000 and 100 PFU of WNV Eg101 elicited robust WNV‑specific antibodies responses. Anti-WNV IgM first appeared at day 6, peaked at day 9 and then gradually decreased at days 16 and 21 after infection (Figure 3A). Similarly, anti-WNV IgG first appeared at day 6 and gradually increased up to day 21 after infection (Figure 3B). Titers of both IgM and IgG antibodies were comparable in mice infected with 1000 and 100 PFU of WNV Eg101 (Figure 3A,B).

### 3.4. WNV Eg101 Infection Induces Cross-Reactive Antibodies that Neutralize the WNV NY99 Strain

**Figure 4:**
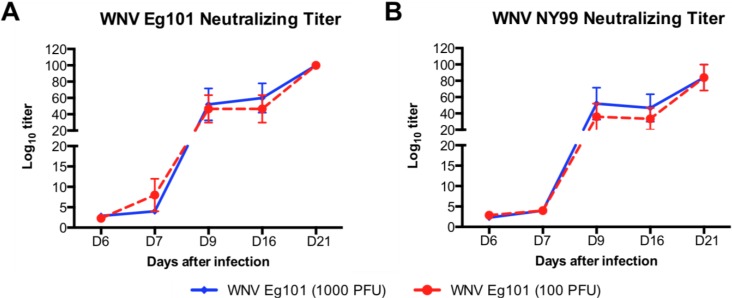
Neutralizing antibodies titers in the serum of WNV Eg101 infected mice. Serum collected from WNV Eg101 infected mice at indicated time points was serially diluted from 1:40 to 1:5000 and PRNT was conducted against (**A**) WNV Eg101 and (**B**) WNV NY99 viruses. Data are expressed as mean log_10_ titer ±SEM and is representative of two independent experiments (n = 8–16 mice per group).

Serum from WNV Eg101 infected mice was tested for neutralizing antibodies against WNV Eg101 and WNV NY99 strains using PRNT assay. High titers of neutralizing antibodies to WNV Eg101 and WNV NY99 strains were detected in serum of WNV Eg101 infected mice at days 6, 7, 9, 16 and 21 after infection (Figure 4A,B). Serum from WNV Eg101 infected mice neutralized WNV Eg101 and WNV NY99 strains with similar efficiency. Moreover, titers of neutralizing antibodies were similar in mice infected with 1000 and 100 PFU of WNV Eg101 (Figure 4A,B).

### 3.5. Protection of WNV Eg101 Immunized Mice against Subcutaneous or Intracranial WNV NY99 Challenge

Three weeks after infection, PBS (Mock) and WNV Eg101 immunized mice were challenged subcutaneously or intracranially with lethal dose (1000 PFU) of WNV NY99 and the disease symptoms and mortality were observed for 21 days. As expected, subcutaneous or intracranial challenge of PBS-infected mice resulted in 94% and 100% mortality, respectively (Table 1). In comparison, WNV Eg101 immunized mice were completely protected against WNV disease after both subcutaneous and intracranial challenge of WNV NY99 and demonstrated no morbidity and mortality (Table 1). During the 21 days observation period after the challenge, none of WNV Eg101 immunized mice displayed any signs of disease, such as immobility, ruffled fur, or anorexia, while all mice in the PBS-infected group demonstrated severe symptoms and succumbed to paralytic encephalitis between 8 and 11 days after challenge (Table 1).

We further determined the virus loads in WNV Eg101 immunized mice at days 1, 3, 8, and 14 after subcutaneous challenge with WNV NY99. No virus was detected in the serum of any mice at days 1, 3, 8 and 14 after challenge.

**Table 1:** Protection of immunized mice from lethal challenge with WNV NY99*

	Subcutaneous challenge with WNV NY99 (1000 PFU)^#^			Intracranial challenge with WNV NY99 (1000 PFU)^#^	
Mouse group	% Morbidity (sick/challenged)	% Mortality (dead/challenged)		% Morbidity (sick/challenged)	% Mortality (dead/challenged)
PBS	100 (16/16)	94 (15/16)		100 (16/16)	100 (16/16)
WNV Eg101 (1000 PFU)	0 (0/16)	0 (0/16)		0 (0/16)	0 (0/16)
WNV Eg101 (100 PFU)	0 (0/16)	0 (0/16)		0 (0/16)	0 (0/16)

* Mice were challenged three-weeks after immunization. ^#^ Mice were observed for 21 days after challenge.

## 4. Discussion

In this study, we demonstrated that immunization with the live and infectious WNV Eg101 strain, which is non-virulent in adult mice, induces potent immune responses and completely protects mice against both subcutaneous and intracranial challenge with a lethal dose of highly virulent WNV NY99 strain. Infection of mice with both 1000 and 100 PFU of WNV Eg101 induced high titers of WNV specific IgM and IgG antibodies, and cross-reactive neutralizing antibodies, which were detectable as early as day 6 after infection and neutralized both WNV Eg101 and WNV NY99 strains with similar efficiency.

### 4.1. Eg101 Strain Is Non-Pathogenic Compared to NY99 Strain of WNV

Despite the high degree of homology between the two strains, it has been demonstrated that WNV Eg101 is significantly less virulent in adult mice than the WNV NY99 by peripheral inoculation [25,35]. In Egypt, WNV Eg101 is widespread and endemic in some areas. However, overt infection in humans is extremely rare and non-fatal, demonstrating natural attenuation of this strain in humans [24]. Similarly, our data also demonstrate that WNV Eg101 is largely non-pathogenic in adult mice. We did not observe any morbidity and mortality in mice infected with 100 PFU of WNV Eg101. While similar dose of WNV NY99 resulted in high morbidity and mortality in mice. Moreover, 1000 PFU dose, which is almost 100% fatal in WNV NY99 infected mice, only resulted in 6% mortality in WNV Eg101 infected mice. Low mortality in WNV Eg101 infected mice was also associated with reduced WNV replication in the serum. The viremia in majority of Eg101 infected mice was significantly lower and short-lived than in WNV NY99 infected mice. These data suggest that infection with WNV Eg101 is associated with limited virus replication resulting in low pathogenicity.

### 4.2. WNV Eg101 Infection in Mice Induced Strong WNV-Specific Antibodies Responses

The induction of high-titer WNV-specific and neutralizing antibodies after infection has been demonstrated as a primary mechanism of protection during secondary challenge [19,36]. Therefore, the development of a WNV-specific humoral immune response is an important criterion for the development of an effective vaccine. Following infection with WNV Eg101 in mice, we observed high WNV-specific IgM, IgG and neutralizing antibodies titers. High titers of WNV-specific antibodies were developed as early as 6 days after infection and remained elevated for the entire duration of the study. Although, WNV Eg101 is genetically and antigenically very closely related to the WNV NY99, the neutralizing antibodies response to WNV Eg101 and its cross-reactivity to WNV NY99 is not well documented [8,25]. In this study, we have demonstrated for the first time that WNV Eg101 infected mice developed antibodies responses, which is cross-reactive and neutralized both strains with similar efficiency. Despite the differences in the survival and virus titers, mice infected with 1000 and 100 PFU of WNV Eg101 developed similar antibodies responses.

### 4.3. WNV Eg101 Immunized Mice Were Completely Protected against Pathogenic WNV NY99

Previous studies have established that *in vitro* neutralization activity of anti-WNV antibodies correlates with *in vivo* protection [13,14]. Moreover, it has been demonstrated that passive immunization with neutralizing monoclonal antibodies provided protection against WNV challenge [13,14,16]. Similarly in our study, high cross-reactive neutralizing antibodies titers in WNV Eg101 immunized mice provided complete protection against lethal challenge with WNV NY99. Mice immunized with both 1000 and 100 PFU of WNV Eg101 demonstrated 100% protection after challenge with lethal dose of WNV NY99. Interestingly, we also observed complete protection after direct inoculation of WNV NY99 into the brain. It has been demonstrated that T cell mediated immunity is essential to clear WNV in the brain after primary infection [17,18,19]. In this study, we have not analyzed the cellular immune response to WNV Eg101 infection. However, it has been demonstrated that a robust neutralizing antibodies response is sufficient to prevent lethal infection after both peripheral and intracranial challenge with WNV NY99 regardless of the presence of CD8^+^ T cells [19]. Therefore, protection against challenge in WNV Eg101 immunized mice could be due to alone high antibodies responses or both antibodies and cellular responses. Moreover, our inability to detect virus in serum after challenge suggests the complete inhibition of virus replication mediated by high pre-exposure cross-neutralizing antibodies titers against WNV.

## 5. Conclusions

We demonstrate for the first time the cross protection to WNV NY99 after immunization with non-virulent WNV Eg101 strain. Several classes of candidate WNV vaccines such as inactivated, subunit, chimeric and live-attenuated vaccines have been developed [19,36]. Although attenuated and inactivated WNV vaccines are currently in use for veterinary purpose, no vaccine is approved for human use [22]. Live-attenuated vaccines derived from attenuated strains such as Kunjin virus have been demonstrated to protect mice from lethal challenge with a virulent WNV strain [20,21,36]. Data presented in this study provides proof-of-concept for the use of WNV Eg101 as an alternative to currently sought vaccine candidates for development of an attenuated WNV vaccine. However, additional studies are warranted to assess the role of cellular immunity in mediating protection and if attenuation of non-virulent WNV Eg101 strain will also provide similar levels of protection against lethal challenge with virulent WNV strains.

## References

[1] Hayes E.B., Gubler D.J. (2006). West Nile virus: Epidemiology and clinical features of an emerging epidemic in the United States. Annu. Rev. Med..

[2] Smithburn K., Hughes T., Burke A., Paul J. (1940). A neurotropic virus isolated from the blood of a native of Uganda. Am. J. Trop. Med. Hyg..

[3] Hubalek Z., Halouzka J. (1999). West Nile fever—A reemerging mosquito-borne viral disease in Europe. Emerg. Infect. Dis..

[4] Beasley D.W., Barrett A.D., Tesh R.B. (2013). Resurgence of West Nile neurologic disease in the United States in 2012: What happened? What needs to be done?. Antivir. Res..

[5] Donadieu E., Bahuon C., Lowenski S., Zientara S., Coulpier M., Lecollinet S. (2013). Differential virulence and pathogenesis of West Nile viruses. Viruses.

[6] Murray K.O., Mertens E., Despres P. (2010). West Nile virus and its emergence in the United States of America. Vet. Res..

[7] Lanciotti R.S., Roehrig J.T., Deubel V., Smith J., Parker M., Steele K., Crise B., Volpe K.E., Crabtree M.B., Scherret J.H. (1999). Origin of the West Nile virus responsible for an outbreak of encephalitis in the northeastern United States. Science.

[8] Lanciotti R.S., Ebel G.D., Deubel V., Kerst A.J., Murri S., Meyer R., Bowen M., McKinney N., Morrill W.E., Crabtree M.B. (2002). Complete genome sequences and phylogenetic analysis of West Nile virus strains isolated from the United States, Europe, and the Middle East. Virology.

[9] Petersen L.R., Carson P.J., Biggerstaff B.J., Custer B., Borchardt S.M., Busch M.P. (2013). Estimated cumulative incidence of West Nile virus infection in US adults, 1999–2010. Epidemiol. Infect..

[10] Rizzo C., Salcuni P., Nicoletti L., Ciufolini M.G., Russo F., Masala R., Frongia O., Finarelli A.C., Gramegna M., Gallo L. (2012). Epidemiological surveillance of West Nile neuroinvasive diseases in Italy, 2008 to 2011. Euro. Surveill..

[11] Papa A. (2012). West Nile virus infections in Greece: An update. Expert Rev. Anti. Infect. Ther..

[12] Calistri P., Giovannini A., Hubalek Z., Ionescu A., Monaco F., Savini G., Lelli R. (2010). Epidemiology of west nile in europe and in the mediterranean basin. Open Virol. J..

[13] Diamond M.S., Shrestha B., Marri A., Mahan D., Engle M. (2003). B cells and antibody play critical roles in the immediate defense of disseminated infection by West Nile encephalitis virus. J. Virol..

[14] Diamond M.S., Sitati E.M., Friend L.D., Higgs S., Shrestha B., Engle M. (2003). A critical role for induced IgM in the protection against West Nile virus infection. J. Exp. Med..

[15] Diamond M.S., Pierson T.C., Fremont D.H. (2008). The structural immunology of antibody protection against West Nile virus. Immunol. Rev..

[16] Ben-Nathan D., Lustig S., Tam G., Robinzon S., Segal S., Rager-Zisman B. (2003). Prophylactic and therapeutic efficacy of human intravenous immunoglobulin in treating West Nile virus infection in mice. J. Infect. Dis..

[17] Shrestha B., Diamond M.S. (2004). Role of CD8+ T cells in control of West Nile virus infection. J. Virol..

[18] Sitati E.M., Diamond M.S. (2006). CD4+ T-cell responses are required for clearance of West Nile virus from the central nervous system. J. Virol..

[19] Shrestha B., Ng T., Chu H.J., Noll M., Diamond M.S. (2008). The relative contribution of antibody and CD8+ T cells to vaccine immunity against West Nile encephalitis virus. Vaccine.

[20] Yamshchikov G., Borisevich V., Seregin A., Chaporgina E., Mishina M., Mishin V., Kwok C.W., Yamshchikov V. (2004). An attenuated West Nile prototype virus is highly immunogenic and protects against the deadly NY99 strain: a candidate for live WN vaccine development. Virology.

[21] Monath T.P., Liu J., Kanesa-Thasan N., Myers G.A., Nichols R., Deary A., McCarthy K., Johnson C., Ermak T., Shin S. (2006). A live, attenuated recombinant West Nile virus vaccine. Proc. Natl. Acad. Sci. USA.

[22] Ng T., Hathaway D., Jennings N., Champ D., Chiang Y.W., Chu H.J. (2003). Equine vaccine for West Nile virus. Dev. Biol..

[23] Melnick J.L., Paul J.R., Riordan J.T., Barnett V.H., Goldblum N., Zabin E. (1951). Isolation from human sera in Egypt of a virus apparently identical to West Nile virus. Proc. Soc. Exp. Biol. Med..

[24] Hurlbut H.S., Rizk F., Taylor R.M., Work T.H. (1956). A study of the ecology of West Nile virus in Egypt. Am. J. Trop. Med. Hyg..

[25] Shirato K., Kimura T., Mizutani T., Kariwa H., Takashima I. (2004). Different chemokine expression in lethal and non-lethal murine West Nile virus infection. J. Med. Virol..

[26] Kumar M., Roe K., Nerurkar P.V., Namekar M., Orillo B., Verma S., Nerurkar V.R. (2012). Impaired virus clearance, compromised immune response and increased mortality in type 2 diabetic mice infected with west nile virus. PLoS One.

[27] Kumar M., Roe K., Orillo B., Muruve D.A., Nerurkar V.R., Gale M., Verma S. (2013). Inflammasome adaptor protein Apoptosis-associated speck-like protein containing CARD (ASC) is critical for the immune response and survival in west Nile virus encephalitis. J. Virol..

[28] Roe K., Kumar M., Lum S., Orillo B., Nerurkar V.R., Verma S. (2012). West Nile virus-induced disruption of the blood-brain barrier in mice is characterized by the degradation of the junctional complex proteins and increase in multiple matrix metalloproteinases. J. Gen. Virol..

[29] Kumar M., Roe K., Nerurkar P.V., Orillo B., Thompson K., Verma S., Nerurkar V.R. (2014). Reduced immune cell infiltration and increased pro-inflammatory mediators in the brain of type 2 diabetic mouse model infected with West Nile virus. J. Neuroinflammation.

[30] Kumar M., Nerurkar V.R. (2014). Integrated analysis of microRNAs and their disease related targets in the brain of mice infected with West Nile virus. Virology.

[31] Kumar M., Verma S., Nerurkar V.R. (2010). Pro-inflammatory cytokines derived from West Nile virus (WNV)-infected SK-N-SH cells mediate neuroinflammatory markers and neuronal death. J. Neuroinflammation.

[32] Namekar M., Kumar M., O'Connell M., Nerurkar V.R. (2012). Effect of Serum Heat-Inactivation and Dilution on Detection of Anti-WNV Antibodies in Mice by West Nile Virus E-protein Microsphere Immunoassay. PLoS One.

[33] Lieberman M.M., Nerurkar V.R., Luo H., Cropp B., Carrion R., de la Garza M., Coller B.A., Clements D., Ogata S., Wong T., Martyak T., Weeks-Levy C. (2009). Immunogenicity and protective efficacy of a recombinant subunit West Nile virus vaccine in rhesus monkeys. Clin. Vaccine Immunol..

[34] (2014). GraphPad Prism.

[35] Weiner L.P., Cole G.A., Nathanson N. (1970). Experimental encephalitis following peripheral inoculation of West Nile virus in mice of different ages. J. Hyg. (Lond.).

[36] Pinto A.K., Richner J.M., Poore E.A., Patil P.P., Amanna I.J., Slifka M.K., Diamond M.S. (2013). A hydrogen peroxide-inactivated virus vaccine elicits humoral and cellular immunity and protects against lethal West Nile virus infection in aged mice. J. Virol..

